# On Some Diseases of the Nervous System in Childhood

**Published:** 1871-09

**Authors:** 


### Book Notices.

On Some Diseases of the Nervous System in Childhood: being the Lumlian Lectures Delivered at the Royal College of Physicians of London, in March, 1871. By Charles West, M.D., Fellow and Senior Censor of the College; Physician to the Hospital for Sick Children. Philadelphia: Henry C. Lea. 1871.

This is a neatly published duodecimo volume of 131 pages. It is made up of three lectures, the first, on Neuralgia and Epilepsy; the second, on Chorea and Paralysis; and the third, on Disorder and Loss of Power of Speech, Mental and Moral Peculiarities and their Disorders. The author is one of the most experienced and able writers on the diseases of women and children that we have in the profession; and the present little volume will be found worthy of perusal by every practitioner.

